# Genome Assembly of *Cordia subcordata*, a Coastal Protection Species in Tropical Coral Islands

**DOI:** 10.3390/ijms242216273

**Published:** 2023-11-13

**Authors:** Yi-Lan Chen, Zheng-Feng Wang, Shu-Guang Jian, Hai-Min Liao, Dong-Ming Liu

**Affiliations:** 1 Innovation Academy of South China Sea Ecology and Environmental Engineering, South China Botanical Garden, Chinese Academy of Sciences, Guangzhou 510650, China; 2 Key Laboratory of Karst Georesources and Environment, Ministry of Education, College of Resources and Environmental Engineering, Guizhou University, Guiyang 550025, China; 3 Collaborative Innovation Center for Mountain Ecology & Agro-Bioengineering, College of Life Sciences/Institute of Agro-Bioengineering, Guizhou University, Guiyang 550025, China; 4 Guangdong Provincial Key Laboratory of Applied Botany, South China Botanical Garden, Chinese Academy of Sciences, Guangzhou 510650, China; 5 Key Laboratory of Vegetation Restoration and Management of Degraded Ecosystems, Key Laboratory of Carbon Sequestration in Terrestrial Ecosystem, South China Botanical Garden, Chinese Academy of Sciences, Guangzhou 510650, China

**Keywords:** *Cordia subcordata*, tropical coral islands, sequencing, genome assembly, gene annotation, phylogenetic analysis

## Abstract

*Cordia subcordata* trees or shrubs, belonging to the Boraginaceae family, have strong resistance and have adapted to their habitat on a tropical coral island in China, but the lack of genome information regarding its genetic background is unclear. In this study, the genome was assembled using both short/long whole genome sequencing reads and Hi–C reads. The assembled genome was 475.3 Mb, with 468.7 Mb (99.22%) of the sequences assembled into 16 chromosomes. Repeat sequences accounted for 54.41% of the assembled genome. A total of 26,615 genes were predicted, and 25,730 genes were functionally annotated using different annotation databases. Based on its genome and the other 17 species, phylogenetic analysis using 336 single-copy genes obtained from ortholog analysis showed that *C. subcordata* was a sister to *Coffea eugenioides*, and the divergence time was estimated to be 77 MYA between the two species. Gene family evolution analysis indicated that the significantly expanded gene families were functionally related to chemical defenses against diseases. These results can provide a reference to a deeper understanding of the genetic background of *C. subcordata* and can be helpful in exploring its adaptation mechanism on tropical coral islands in the future.

## 1. Introduction

**Figure 1:**
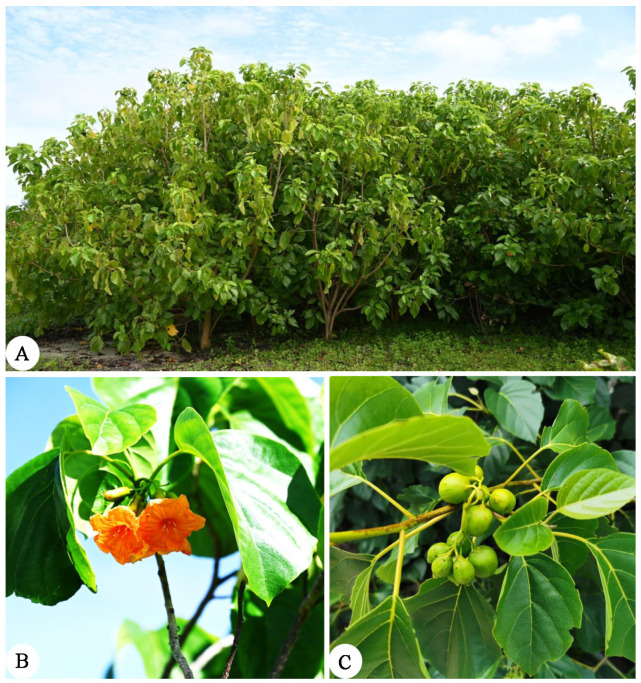
*Cordia subcordata*. (**A**) Whole tree, (**B**) flowers, and (**C**) fruits. Photographed by D.-M. Liu.

*Cordia subcordata* (2n = 2x = 32) trees or shrubs (Figure 1) belong to the Boraginaceae family [1]. Most species of the Boraginaceae family are herbaceous, but all species from the genus *Cordia* are woody; thus, there are special groups for their physical forms [2]. The genus *Cordia* has 250–300 species around the world, mainly distributed on the east coast of Africa, India, Vietnam, and the southern Pacific islands [3]. In China, there are just six species of the genus *Cordia*, namely *C. cochinchinensis*, *C. cumingiana*, *C. dichotoma*, *C. furcans*, *C. myxa*, and *C. subcordata*, which are mostly distributed in southwest and southern China and are mainly found in Hainan province [4]. Most species of the genus *Cordia* have special chemical, biological, and pharmacological properties, which have a high research value in ethnobotany and ethnopharmacology. In addition, their secondary chemicals have a variety of applications in many aspects, such as anti-inflammation, wound healing, de-worming, anti-malaria, diuretics, and treating pulmonary diseases [5].

The tropical coral islands of China have a typical tropical ocean monsoon climate, with long sunshine durations, strong radiation, and high temperature all year around [6]. The soils are strongly alkaline and rich in calcium and phosphorus [1,7]. *C. subcordata* is a dominant evergreen small tree found on the tropical coral islands of China. It has a strong resistance to wind and dust because of its developed root systems. A physiological ecology study revealed that its characteristics, including a large leaf area, a high leaf epidermal stomata density, a thick upper epidermis, a low specific leaf area value, a high vessel diameter and density, low leaf water conductivity, and high xylem density, could favor *C. subcordata* in fully utilizing water and adapting to the poor soil, high temperatures, strong sunlight, and drought conditions [8]. In the field, *C. subcordata* is also observed to be pest- and pathogen-resistant (personal observation by D.-M. Liu). This could be attributed to the phytoconstituents in its tissues. In fact, it has been reported that the *Cordia* species contains various chemical components, such as quinones, terpenoid, steroids, alkaloids, flavonoids, and/or saponins [5,9], in their leaves, stem, root, fruits, or seeds. These chemical components not only have medicinal value (see above), but are also widely used as weapons against pests and pathogens in plants in nature. For example, *Cordia sebestena* is a widely planted tree in the area of the tropics [10]. It has long been used as a traditional medicinal plant and has also been found to be pest- and disease-resistant, due to the chemical composition of many parts of the plant (leaves, flower, and bark). Therefore, given its high levels of environmental adaptation, *C. subcordata* could be used for artificial plant community construction, vegetation restoration, and the improvement of the environmental conditions of tropical coral islands [8,11].

Moreover, *C. subcordata* could have more than medical and ecological value. A pollen morphology study on the genus *Cordia* in Boraginaceae showed that its pollen morphologies were diverse and unique, displaying rather primitive characteristics [2], which exhibited the fact that the *Cordia* species also has a high scientific research value. Specifically, reproductive and pollination biological studies showed that *C. subcordata* was a typical distylous species, and such distyly is rare on oceanic islands [12].

Due to the anthropogenic disturbances of coral islands, the populations of *C. subcordata* have declined and have been threatened. Currently, it is listed as a second-level national key protected wild plant in China (https://www.gov.cn/zhengce/zhengceku/2021-09/09/content_5636409.htm, accessed on 19 September 2023). Considering its low seedling regeneration rate in nature, Xiong et al. [13] successfully established a tissue culture protocol, using young stem sections with apical shoot buds, for the mass propagation of *C. subcordata*, which provided a powerful tool for preserving this endangered plant species and may lead to its future usage in reforestation and coral island preservation.

However, previous research on *C. subcordata* has mainly focused on its biological properties and morphological structures but does not include genome or related genetic studies. Since the discovery of the DNA double helix structure, many scientists have put a lot of effort into determining the sequence of genomes, and more and more efficient sequencing technologies have been designed to accurately sequence genomes. With the increasing recognition of high-quality genome sequences, the assisted assembly technology for chromosome-level genomes has emerged [14]. In recent years, chromosome-scale genomes have been reported in numerous plant species, which provide insights into their genetic basis and population genetic structure [15,16,17]. In this study, we sequenced the whole genome and transcriptome of *C. subcordata* with the BGISEQ (BGI-Shenzhen, Shenzheng, China) and OXFORD NANOPORE (Oxford Nanopore Technologies, Oxford, United Kingdom) sequencing platforms and assembled and annotated its whole genome. Additionally, based on this genome, orthologous gene identification and phylogenetic analysis were performed. This deep insight into the genetic background of *C. subcordata* could be helpful in exploring its adaptation mechanisms for high temperature, high salinity, strong alkalinity, and other habitats in tropical coral islands, and in laying a foundation for germplasm conservation and genetic breeding.

## 2. Results

### 2.1. Genome Sequencing and Assembly

In this study, the BGISEQ and OXFORD NANOPORE sequencing platform produced approximately 64.3 Gb short whole genome sequencing (WGS) reads, 125.0 Gb long WGS reads, 75.3 Gb Hi-C reads, and 8 Gb RNA-seq reads.

**Figure 2:**
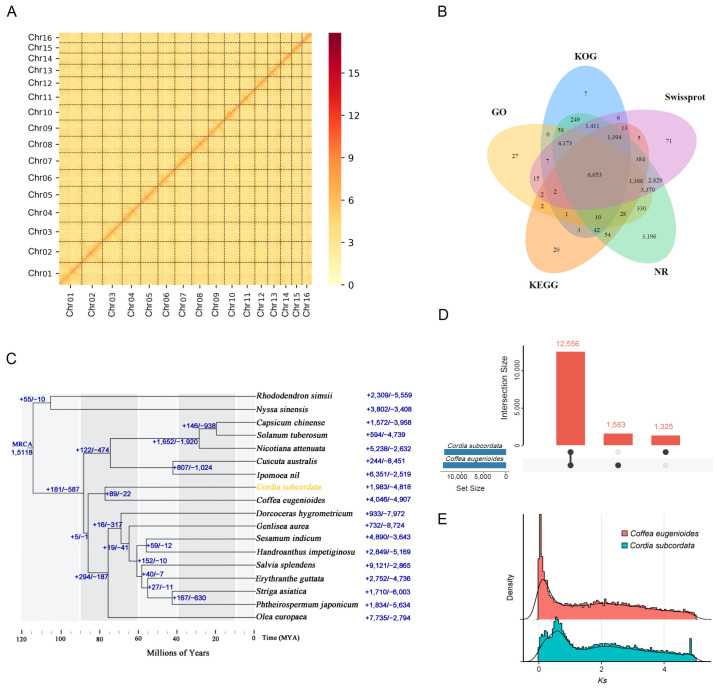
Genome assembly, annotation, and phylogenetic analysis of *Cordia subcordata*. (**A**) Hi-C interaction heat map (bin length 10,000 bp), (**B**) Venn graph of annotation in five databases, (**C**) phylogenetic tree with mapped gene family expansions (+) and contractions (–) in *C. subcordata a*nd other species, (**D**) shared gene families between *C. subcordata* and *Coffea eugenioides*, (**E**) whole genome duplication analysis with comparisons of the synonymous substitutions per synonymous site (Ks) value in *C. subcordata* and *C.* *eugenioides.*

The genome size was initially evaluated to be 524,294,375 bp, using distribution analysis, with a k-mer of 17 and short WGS reads. K-mer analysis also showed that this genome had a heterozygosity of 0.07% and a repetitive sequence content of 42.1%. The primarily assembled genome was 475,321,542 bp, with 92 contigs and a contig N50 length of 16,399,519 bp (Table 1). To obtain high-quality assembly, the contigs were corrected and scaffolded using Hi-C reads. Finally, the assembled genome was 470,353,539 bp in length, with 468,670,648 bp (99.22%) of sequences assembled into 16 chromosomes, and the chromosomes ranged in size from 18,188,694 bp (Chromosome 1) to 42,352,411 bp (Chromosome 16) (Figure 2A, Table S1).

### 2.2. Completeness of the Genome and Quality Evaluation

BUSCO analysis indicated that 95.28% of the core genes were completely captured by the genome assembly, which included 90.49% complete and single-copy and 4.79% complete and duplicated genes, while 0.97% were captured as fragments, and 3.75% were missing (Table 2). Additionally, a simple assessment of genome integrity, by mapping short WGS reads to the assembled genome, indicated that 97.90% of the reads were properly mapped. These evaluations implied a high completeness of the *C. subcordata* genome assembly.

### 2.3. Repeat Sequence Prediction

A total of 256,983,673 bp sequences were identified as repetitive elements by different repeat-identifying programs, accounting for 54.41% of the assembled genome (Table S2). The most abundant repetitive elements were transposable element (TE) sequences, with 241,514,515 bp (51.13%), followed by unknown-type repeats, with 10,733,217 bp (2.27%). Within TE sequences, the most abundant were terminal repeats (LTRs), with 167,223,552 bp (35.4%). The tandem repeats comprised 4,121,922 bp (0.87%), and SSRs comprised 219,915 bp (0.05%) in the assembled genome.

### 2.4. Gene Prediction and Annotation

A total of 26,615 protein-coding genes were predicted in the *C. subcordata* assembled genome. The average gene length was 4074 bp, and the average coding sequence (CDS) length was 1268 bp. Each predicted gene contained 5.4 exons, with a mean sequence length of 236 bp (Table S3). In addition, 3010 non-coding RNAs, including 928 (0.015%) tRNAs, 282 (0.056%) rRNAs, 90 (0.003%) miRNAs, and 297 (0.004%) snRNAs, were predicted in the *C. subcordata* assembled genome (Table S4). Overall, 25,730 genes (96.67%) were functionally annotated in at least one database (Figure 2B), including 21,703 (81.54%) in the Swiss-Prot protein database, 9981 (37.50%) in the Kyoto Encyclopedia of Gene and Genomes (KEGG) database, 14,029 (52.71%) in the Eukaryotic Orthologous Groups of protein (KOG) database, 16,046 (60.29%) in the Gene Ontology (GO) database, and 25,549 (95.99%) in the Non-Redundant Protein (NR) database (Table 3).

### 2.5. Orthologous Gene Identification and Phylogenetic Analysis

A total of 614,770 genes from 18 species were used for orthologous gene group identification, and 30,853 orthologous gene groups were obtained using Orthofinder. For *C. subcordata*, 25,078 genes were assigned to 13,881 orthologous gene groups, and 217 orthologous gene groups contained 737 genes specific to *C. subcordata* (Table S5). The phylogenetic tree constructed using 336 single-copy genes resolved that *C. subcordata* was a sister to *Coffea eugenioides*, and the divergence time between them was estimated to be 77 MYA (Figure 2C). Both species shared 12,556 gene families, and 1583 were *C. subcordata* specific (Figure 2D).

Gene family evolution analysis indicated that 1983 gene families exhibited expansion, and 4818 families exhibited contraction in *C. subcordata*. For expanded and contracted gene families in *C. subcordata*, 51 gene families, consisting of 639 genes, were significantly expanded (*p* < 0.05), and 44 gene families, consisting of 72 genes, were significantly contracted. Enrichment analysis showed that the significantly expanded gene families were mainly related to the purine, pigment, and lipid metabolic/biosynthetic process in the GO biological process category (Table S6), and to isoquinoline alkaloid biosynthesis, terpenoid backbone biosynthesis, tyrosine metabolism, and ion channels in KEGG (Table S7). The significantly contracted gene families were mainly related to protein phosphorylation in the GO biological process category (Table S8), and to the MAPK signaling pathway, plant–pathogen interaction, and signal transduction in KEGG (Table S9).

### 2.6. Whole Genome Duplication and Gene Duplication

Whole genome duplication (WGD) analysis indicated that *C. subcordata* underwent two round WGD events (Figure 2E), and the most ancient one was shared with *C. eugenioides*, its sister species in the phylogeny analysis (Figure 2C).

Gene duplications indicated that there were 10,309 WGD-type genes, 2902 tandem duplications (TD)-type genes, 1033 proximal duplications (PD)-type genes, 49 transposed duplications (TRD)-type genes and 8044 dispersed duplications (DD)-type genes in *C. subcordata*. Enrichment analysis indicated that WGD-related genes in *C. subcordata* are mainly associated with monoatomic ion transport, DNA-templated transcription, and cell walls in the GO biological process category (Table S10), and with G protein-coupled receptors, phagosome, and the photosynthesis of antenna proteins in KEGG (Table S11). Enrichment analysis indicated the TD-related genes in *C. subcordata* are mainly associated with defense response and amino sugar catabolic processes in the GO biological process category (Table S12), and with terpenoid biosynthesis, stilbenoid, diarylheptanoid and gingerol biosynthesis, and isoquinoline alkaloid biosynthesis in KEGG (Table S13). Enrichment analysis indicated that PD-related genes in *C. subcordata* are mainly associated with tricarboxylic acid metabolic process in GO biological process category (Table S14), and with the biosynthesis of various plant secondary metabolites and isoquinoline alkaloid biosynthesis in KEGG (Table S15).

## 3. Discussion

Boraginaceae is the only family in Boraginales that is a core group and is one of the largest groups within the lamiids clade, which is rarely published in genome aspects, so the genome of *C. subcordata* is important for the Boraginaceae family. Generally, Boraginaceae can be classified into four subfamilies: Boraginaceae, Heliotropiaceae, Ehretiaceae, and Cordiaceae [18,19]. However, due to the rapid evolutionary divergence within the Boraginaceae family, the phylogenetic relationships in the family remain elusive [19]. The Cordiaceae subfamily, in addition to *Cordia*, may also include the *Varronia* and *Patagonula* genera [19,20], which means the species in these two genera are the most genetically close to *Cordia*. Cohen [18] conducted both morphological and molecular phylogenetic studies on the Boraginaceae family. Based on combined morphological and molecular markers (including *mat*k, *ndh*F, and *trn*L-*trn*F markers of cpDNA), his results showed that Cordiaceae, Heliotropiaceae, and Ehretiaceae were more phylogenetically closed and formed a clade, but, in the clade, their relationships could not be further resolved [18]. Moreover, his results using only molecular markers (including *mat*k, *ndh*F, and *trnL-trn*F markers of cpDNA, and ITS markers of nrDNA) indicated that Cordiaceae is the most closed to Heliotropiaceae. Nevertheless, further studies using *rbc*L, *ndh*F, and *trn*L-*trn*F markers of cpDNA [19] and whole cpDNA genomes [20] indicated that Cordiaceae is sister to Ehretiaceae. Since no *Varronia*, *Patagonula*, Ehretiaceae, or Heliotropiaceae genomes are available currently, we could not include them in our phylogeny analysis. Therefore, we expanded our comparative genomic analysis to include more species in Lamiids, as their genomes have been assembled and annotated, which resulted in 15 Lamiids species in 11 families, allowing us to accurately construct the phylogeny of our studied species. Our phylogenetic analysis indicated that *C. subcordata* was sister to *Coffee eugenioides*, when none of the other Boraginaceae species were included. This result was consistent with the results of Alshegaihi et al. [21]. In their study, they assembled the complete cpDNA genome of *C. monoica*. Their phylogeny analysis, using the *C. monoica* cpDNA genome, indicated that Boraginaceae was sister to Rubiaceae (*Coffea arabica* used in their study).

Complete and accurate genome assembly is generally hindered by the large genome size (>1 Gb), high heterozygosity (>0.5%), and repetitive sequences (>50%) in all genomes [22]. The genome features in this study, discovered by the k-mer method with short WGS reads, indicated that *C. subcordata* has an intermediate genome size, low heterozygosity, and repetitive sequences, which facilitated its assembling, which in turn was especially aided with long-read sequencing technology. In this study, the high-quality 470.35 Mb chromosome-level genome of *C. subcordata* was obtained using the Hi-C reads to improve genome assembly. Its genome size was larger than *Echium plantagineum* (351.50 Mb) and *Lithospermum erythrorhizon* (367.41 Mb), which are reported to be in the family Boraginaceae [23,24]. However, compared to the genome assembly of *E*. *plantagineum* and *L. erythrorhizon*, the predicted protein-coding genes (26,615) in *C. subcordata* were smaller than both of the above, i.e., 42,316 in *E*. *plantagineum* [23] and 27,720 in *L. erythrorhizon* [24].

A comprehensive genome size estimation conducted for thirty-eight taxa (274 individuals) in the Boraginaceae family from the Czech Republic using flow cytometry revealed that the lowest genome size in the family was about 274 Mb and the largest was 16 Gb, and that most species had a size between 500 Mb and 1.5 Gb [25]. Therefore, *C. subcordata*, as well as the *E*. *plantagineum* and *L. erythrorhizon* mentioned above, had a relatively small genome size for a member of the Boraginaceae family. A study done by Kobrlova and Hrones [25] also indicated that the genome sizes in the Boraginaceae family were correlated with its life history, ecology, and phylogeny. Perennial plants and species living in natural habitats harbored relatively larger genome sizes in the Boraginaceae family.

Chemical components are accumulated in different parts in different Boraginaceae species [7,8]. For example, alkannin/shikonin are distinctive secondary metabolites and commonly found in the root periderm of *E. plantagineum* and *L. erythrorhizon* [23,24]. The genomes of these two species were used to reveal the alkannin/shikonin pathway and to find some key genes in this pathway. Using CAFE, we detected variations in gene family size, resulting from the gain or loss of genes, which represent the expansion or contraction of the gene family. Expanded gene families are usually created by gene duplication events during selection [26]. Duplicated genes can increase the quantity of protein products, benefiting plant adaptation [27]. We observed both alkaloid- and terpenoid-related gene families which were expanded in *C. subcordata*, and these genes showed TD or PD duplication. TD and PD are central duplication processes related to the biotic and abiotic environmental adaption abilities of plant species [28]. Alkaloid and terpenoid are important chemicals produced by plants. Isoquinoline alkaloids (IQAs) are derived from phenylalanine and tyrosine and constitute one of the largest and most diverse groups of alkaloids. IQAs have only been discovered in a limited number of plant species [29,30] and play a key role in the defense against pathogens and herbivores [31,32,33]. IQAs also exhibit exceptionally important pharmacological activities [30] and therefore have high pharmaceutical and commercial value. Terpenes are important natural products with a wide range of applications in plants [34,35,36], but they most often serve as continuously available passive toxic defenses against biological enemies [35]. The *Cordia* species are distributed widely in the tropical regions [9]. The tropics face constant challenges of diverse pests and diseases, and plant defense chemicals play a very important role in their deterrence [37,38]. In prior reports, various chemical components, including sterols, flavonoids, terpenes, alkaloids, and phenolic acids, have been isolated from members of the *Cordia* species [5,9,39,40,41,42]. A recent study, using *C. subcordata* leaf extracts to evaluate its antioxidant properties, identified abundant chemical components, namely polyphenols [43]. From our genome report, further metabolomic analyses are required to identify the chemical constituents of alkaloids and terpenoids in *C. subcordata*.

Interestingly, these expanded gene families are accompanied by the contraction of other biological enemy defense gene families, such as the MAPK signaling pathway. The MAPK signaling pathway has been demonstrated to activate responses and resistance to plant diseases [44,45,46], and MAPK is also a core mediator for the hypersensitive response and subsequent cell death [45,47]. The contraction of MAPK and the expansion of secondary metabolites gene families could represent a trade-off between active and passive defense, where the utilization of MAPK represents an active approach, while alkaloid and terpenoid, the secondary metabolites, represent a passive approach [48,49,50,51]. Therefore, chemicals, such as alkaloids and terpenoids, in *C. subcordata* may be the main mechanism for disease defense on tropical coral islands, and MAPK may have reduced their role in defense. Keeping the balance between gene family expansion and contraction, which maintains the balanced allocation of energy sources, has also been observed in previous studies [52,53,54]. Considering no relevant studies have been carried out regarding *C. subcordata*, future studies are needed to confirm this in a laboratory setting.

## 4. Materials and Methods

### 4.1. Sequencing

Leaf samples from one *C. subcordata* individual, planted in the South China Botanical Garden, were collected. The individual was propagated by a shoot cut from a plant from Yongxing Island of the Paracel Islands (16°49′53″ N, 112°20′22″ E; China). After total RNA and genomic DNA were extracted from the samples, the sequencing libraries, including short- and long-whole genome sequencing (WGS), transcriptome, and Hi-C sequencing, were constructed and then sequenced using the BGISEQ and OXFORD NANOPORE sequencing platforms (Table 4).

### 4.2. Assembly

Before genome assembly, the genome size of *C. subcordata* was estimated using k-mer method, under a k-mer of 17 and short WGS reads. NextDenovo v1.0 [55] was used to correct the long WGS reads with the parameter of “read_cuoff = 1 k, seed_cutoff = 25 k”. The corrected long WGS reads were then inputted into Smartdenovo v1.0.0 [56], to perform assembly, with the parameters of “-k 21, -j 3000”. After assembly, NextPolish v1.0.1 [57] was used to polish the assembly. LACHESIS software (https://github.com/shendurelab/LACHESIS accessed on 5 November 2023) [58] was further used to cluster the assembled contigs into chromosome groups with Hi-C reads. The evaluated assembly was performed by BUSCO v3.0.1 [59], as well as the mapping of the short WGS against the assembly.

### 4.3. Annotation

Tandem repeats in the assembly were searched using GMATA v 2.2 [60] and the Tandem Repeats Finder (TRF) v 4.07b [61], with default parameters. The transposable elements (TEs) were identified using MITE-hunter [62], LTR_finder [63], ltr_harverst [64], LTR_retriver [65], and RepeatModeler v1.0.11 [66]. These inferred results were then combined and inputted into RepeatMasker v1.331 [67], to obtain whole genome repeat sequences.

Gene structure prediction was combined with ab initio-, homology-, and RNA-seq-based methods. Augustus v 3.3.1 [68] was used for ab initio-based gene prediction, and GeMoMa v1.6.1 [69] was used for homology-based gene prediction. Hisat2 v2.1.0 [70], Stringtie v1.3.4d [71], and PASA v2.3.3 [72] were used for RNA-seq-based gene prediction. Finally, EVM [73] and TransposonPSI [74] were applied to integrate gene prediction results and obtain a consensus gene set. For the predicted genes, the annotation of gene functions was carried out by comparing them with the protein sequences of the NR [75], KEGG [76], KOG [77], Swissport [78], and GO [79] databases. Noncoding RNAs, including rRNA, small RNA, cis-regulatory elements, and tRNA, were identified by combined Infernal [80], tRNAscan-SE [81], and RNAmmer [82] programs and the Rfam [83] database.

### 4.4. Orthologous Gene Group Identification and Whole Genome Duplication Analysis

OrthoFinder 2.4.0 [84,85] was used to identify the orthologous gene groups (i.e., gene families) among *C. subcordata* and the other 17 species (Table S16). These species included 15 Lamiids species in 11 families and two outgroup species (*Rhododendron simsii* and *Nyssa sinensis*). After ortholog group identification, single-copy ortholog sequences were selected by OrthFinder, to perform phylogeny analysis among the species. Based on phylogeny results, species divergence times were estimated by TreePL [86,87] with 5 species pairs (Table 5) used as time calibration points and their estimated divergence time (million years ago, MYA) derived from http://timetree.org/. Then, using the phylogenic tree, containing species divergence time, expansions and contractions of the orthologous gene families were estimated using CAFE v5 [88]. For significantly expanded and contracted gene families, enrichment analyses by GO and the KEGG database were performed using TBtools v1.115 [89]. Ancient whole genome duplication events in *C. subcordata* and its sister species *C. eugenioides* were detected using wgd v1.2 [90]. Gene duplications in *C. subcordata* were examined by Doubletrouble v0.99.1 [91]. These duplications were classified into WGD, tandem duplications (TD), proximal duplications (PD), transposed duplications (TRD), and dispersed duplications (DD) [21]. In the doubletrouble analysis, *Coffea eugenioides* was used as the outgroup species. Finally, for WGD-, TD-, and PD-derived genes, enrichment analysis, according to the GO and KEGG databases, was applied using TBtools.

## 5. Conclusions

In summary, we applied both short- and long-read sequencing technologies to assemble the *C. subcordata* genome. The assembled genome was 470,353,539 bp in length, with 468,670,648 bp (99.22%) of sequences assembled into 16 chromosomes. A total of 26,615 genes were predicted and functionally annotated by various databases. The first reported *Cordia* genome provides important information regarding the phylogeny evolution of this genus and helps explore the adaptive mechanism of *C. subcordata* on tropical coral islands. Moreover, with the genomes of the other Boraginaceae species currently sequenced and available, comparative genome analyses including these other genomes will more clearly address these mechanisms, particularly in elucidating the biosynthesis related to the various chemical components (such as alkaloid and terpenoid) of *C. subcordata*.

## Figures and Tables

**Table 1:** The statistical results of the genome assembly.

Type	Contig Length (bp)	Contig Number
N50	16,399,519	10
N60	15,666,772	13
N70	14,697,627	16
N80	12,626,804	19
N90	10,091,180	23
Longest	42,352,411	1
Total	475,321,542	92

**Table 2:** BUSCO results.

Type	Number	Percent (%)
Complete BUSCOs (C)	1372	95.28
Complete and single-copy BUSCOs (S)	1303	90.49
Complete and duplicated BUSCOs (D)	69	4.79
Fragmented BUSCOs (F)	14	0.97
Missing BUSCOs (M)	54	3.75
Total BUSCO groups searched	1440	100

**Table 3:** The statistics of annotated genes in different databases.

Databases	Number	Percent (%)
Swiss-Prot	21,703	81.54
KEGG	9981	37.50
KOG	14,029	52.71
GO	16,046	60.29
NR	25,549	95.99
Total	25,730	96.67

**Table 4:** The sequencing information of *C. subcordata* genome.

Sequencing Project	Sequencing Platform	Sequence Type	Instrument
Short whole genome sequencing	BGISEQ	PE150	DNBSEQ-T7
Long whole genome sequencing	OXFORD NANOPORE	Long reads	GridION
Short-length transcriptome	BGISEQ	PE150	DNBSEQ-T7
Full-length transcriptome	BGISEQ	PE150	DNBSEQ-T7
Hi-C	BGISEQ	PE150	DNBSEQ-T7

**Table 5:** The reference species’ differentiation times from Timetree.

Species Pair	Taxon 1	Taxon 2	Estimated Divergent Time (Million Years Ago, MYA)
1	*Phtheirospermum* *japonicum*	*Striga asiatica*	39–64
2	*Handroanthus* *impetiginosus*	*Sesamum indicum*	56–73
3	*Coffea eugenioides*	*Cordia subcordata*	75–96
4	*Capsicum chinense*	*Solanum tuberosum*	18.2–31.4
5	*Rhododendron simsii*	*Nyssa sinensis*	105–119

## Data Availability

All the genome and raw sequencing reads described in this article are publicly available in the National Center for Biotechnology Information (NCBI) database under project PRJNA909849. The BGISEQ and OXFORD NANOPORE raw sequencing data are deposited under the accession number SRR23354649, SRR23354648, and SRR23354594, and the genome under the accession number ASM2855475v1. Genome assembly and gene annotation data are available at https://doi.org/10.6084/m9.figshare.21286518 accessed on 5 November 2023.

## Supplementary Materials

The following supporting information can be downloaded at: https://www.mdpi.com/article/10.3390/ijms242216273/s1.
